# Physical activity and risk of breast cancer in premenopausal women

**DOI:** 10.1038/sj.bjc.6601175

**Published:** 2003-08-26

**Authors:** G A Colditz, D Feskanich, W Y Chen, D J Hunter, W C Willett

**Affiliations:** 1 Department of Epidemiology, Harvard School of Public Health, Boston, USA; 2 Channing Laboratory, Department of Medicine, Brigham and Women's Hospital and Harvard Medical School, Boston, MA 02115, USA; 3 The Department of Medical Oncology, Dana Farber Cancer Institute, Boston, MA 02115, USA; 4 Department of Nutrition, Harvard School of Public Health, Boston, MA 02115, USA; 5 The Cancer Epidemiology Program, Dana-Farber/Harvard Cancer Center

**Keywords:** breast cancer, physical activity, prospective cohort study

## Abstract

Physical activity appears to be inversely related to risk of breast cancer, yet the results remain inconsistent. To evaluate this relation among premenopausal women and examine variation in risk according to level of obesity and use of oral contraceptives (OCs), the authors examined data from the Nurses’ Health Study II. During 10 years of follow-up, 849 cases of invasive premenopausal breast cancer were confirmed. Physical activity was assessed by self-report at baseline and during follow-up using a validated questionnaire. Total physical activity was unrelated to risk of breast cancer. Women engaging in ⩾27 metabolic equivalent (MET)-h week^−1^ had a multivariate-adjusted relative risk (RR) of 1.04 (95% confidence interval (CI) 0.82–1.33) compared to those in the <3 MET-h week^−1^ category. Among women with a BMI ⩾30 kg m^−2^, we observed a significant positive dose–response relation (*P*=0.04). Activity was unrelated to breast cancer risk at lower levels of BMI. A test for interaction between activity and BMI (<30, ⩾30 kg m^−2^) was statistically significant (*P*=0.02). Among current OC users, higher activity was associated with a non-significantly lower risk of breast cancer (RR=0.59, 95% CI 0.30–1.16 for ⩾27 *vs* <9 MET-h week^−1^, *P* for linear trend=0.14). These results show no overall association between physical activity and risk of breast cancer among premenopausal women, but suggest that the effect of physical activity could be substantially modified by the underlying degree of adiposity. The potential interactions between physical activity, adiposity, and current use of OCs require further study.

The relation of physical activity to risk of breast cancer has been assessed by the International Agency for Research on Cancer, which concluded that, although studies have not been entirely consistent, the overall results support a reduction in risk with higher levels of activity (International Agency for Research on Cancer, 2002). The majority of studies, however, have been focused on postmenopausal breast cancer. Also, upon review of more than 30 studies, it was noted that the most important time periods in life for activity is not currently known. Furthermore, which activities may offer the greatest protection have not been systematically examined.

In a previous analysis of the Nurses’ Health Study II (NHSII) cohort with 372 cases among predominantly premenopausal women, Rockhill *et al* (1998) observed no association between nonoccupational activity levels in late adolescence or the recent past and risk of breast cancer. We now extend that analysis, adding 477 additional premenopausal cases in an effort to understand better the relation between physical activity and risk of premenopausal breast cancer.

In addition to evaluating the type of physical activity, we examined the relation between activity and breast cancer within subgroups of adiposity and oral contraceptive (OC) use, which we hypothesised, might modify the relationship. Potential health effects of (OCs) were the original focus of the NHS II cohort.

## METHODS

The NHS II began in 1989 when 116 671 female registered nurses between 25 and 42 years of age and living within 14 US states responded to an initial mailed questionnaire. Follow-up questionnaires are sent every 2 years and the response rate has been at least 90% in each cycle. Participants report diagnosed diseases and information on lifestyle and other risk factors for disease. Deaths are detected by reports from the postal service or family members and by a search of the National Death Index (Stampfer *et al*, 1984). This analysis includes the 110 468 premenopausal women who responded to the physical activity questions at baseline and had not reported a previous diagnosis of any cancer other than nonmelanoma skin cancer.

### Breast cancer cases

Over the 10 years of follow-up among the premenopausal women, we obtained medical records from 977 (88%) of the 1106 reported diagnoses of breast cancer prior to 1 June, 1999 and before menopause, defined as cessation of menses due to natural causes, surgery, radiation, or chemotherapy. From the medical records, 761 (78%) were confirmed as invasive cancer and included as cases in this analysis; the other 22% were *in situ* and were excluded. Owing to the high degree of self-reporting accuracy, we included in analysis the 88 reports that were confirmed verbally or in writing by the participant but for which we could not obtain medical records, bringing the final breast cancer case count to 849. The median age at diagnosis was 42 years.

### Activity assessment

In 1989, participants reported their average amount of time spent per week during the previous year in each of eight activities: walking or hiking outdoors, jogging (>10 min mile^−1^), running, bicycling (including stationary machine), racquet sports, lap swimming, calisthenics/aerobics, and other aerobic activity. Although most of these activities were recreational, walking could be nonrecreational. For each activity, women chose one of 10 duration categories that ranged from zero to 11 or more hours per week. Walking pace was also reported as easy, average, brisk, or very brisk. Activity was reassessed in 1991 and 1997.

Each activity on the questionnaire was assigned a metabolic equivalent (MET) score based on the classification by Ainsworth *et al* (1993). The MET score for an activity is defined as the ratio of the metabolic rate associated with that activity divided by the resting metabolic rate. For example, walking at an average pace, jogging, and running were assigned MET scores of 3, 7 and 12, respectively. We calculated MET-h week^−1^ for each activity as the reported hours per week engaged in the activity multiplied by the assigned MET score, and the values from the individual activities were summed for a total MET-h week^−1^. To obtain the best long-term measure of activity, values for total MET-h week^−1^ were cumulatively averaged in analyses; that is, at the beginning of each 2-year follow-up cycle, MET-h week^−1^ was the mean of all MET-h week^−1^ calculated from the questionnaires up to that time. We selected cutpoints for data analysis *a priori* to be in three MET increments because three METs is the score for 1 h of walking and we wanted to be able to describe results from our MET-h analyses in terms of the equivalent amount of walking.

The ability of the questionnaire to assess total activity over the previous year was evaluated in a sample of 151 NHS II women (Wolf *et al*, 1994). Compared with four 7-day activity diaries, the questionnaire underascertained activity by approximately 20%. However, the correlation for total MET-h week^−1^ of activity was 0.62 (95% confidence interval (CI) 0.44–0.75), indicating that the questionnaire is a reasonable tool for categorical ranking. For walking, the primary activity among the women in this cohort, the correlation was 0.70 (95% CI 0.49–0.84) when the past-year activity questionnaire was compared with four past-week questionnaires collected seasonally during the year.

### Other risk factors

Weight, use of OCs, reproductive history, and diagnosis of benign breast disease were reported at baseline in 1989 and updated on each follow-up questionnaire. Age at menarche, height, and weight at age 18 years were reported once in 1989. Alcohol consumption was assessed in 1991 and 1995 from a food frequency questionnaire, and family history (mother or sister) of breast cancer was reported in 1989 and again in 1997. Current body mass index (BMI, kg m^−2^) and BMI at age 18 years were calculated from the reported weights and height (Rimm *et al*, 1990; Troy *et al*, 1995), and weight change was calculated as the difference in weight at age 18 years and the current weight in each 2 years follow-up cycle. Time since last birth was also calculated and updated in each follow-up cycle.

### Statistical analysis

Study participants contributed person-time from the return date of their 1989 questionnaire until menopause, the report of a diagnosis of breast cancer or other cancer (except nonmelanoma skin cancer), death, or the end of follow-up on 1999 June 1. The 110 468 women in this analysis contributed a total of 934 082 person-years. Person-time was allocated to the appropriate category for activity and for each of the risk factors at the beginning of every 2-year follow-up cycle. Breast cancer incidence rates were calculated as the number of cases divided by the person-time in each activity category and relative risks (RR) were the ratio of the rate in each upper category compared with the rate in the lowest or referent category. The Cox proportional hazards models were used to calculate multivariate RRs adjusted for all risk factors (Cox, 1972). The *P*-values for linear trend were determined using continuous activity values in the proportional hazards models. The level of statistical significance in all analyses was *P*<0.05.

## RESULTS

At baseline in 1989, the median total activity among the premenopausal women in the study population was 13 MET-h week^−1^; 9% reported zero or minimal activity (i. e., no more than 15 min week^−1^). Walking, the most popular activity, was reported by 84% of the women and contributed 32% of the total MET-h. Calisthenics/aerobics (19%), other aerobic activity (16%), and biking (16%) were the other major contributors.

More active women had a lower BMI, consumed somewhat more alcohol, and were more likely to be nulliparous than sedentary or less-active women (Table 1

**Table 1:** Breast cancer risk factors according to categories of MET-h per week among the 110 468 premenopausal women in the NHS II study population

	**MET-h/Week^−1^ (median)**				
	**<3 (1.6)**	**3–8.9 (5.8)**	**9–17.9 (12.9) **	**18–26.9 (21.9)**	**⩾27 (41.7)**
Age (years)	38.4	38.5	38.4	38.1	37.5
BMI (kg m^−2^)	26.3	25.7	25.1	24.7	24.1
Height (cm)	164	165	165	165	165
Alcohol intake (g day^−1^)	2.5	2.8	3.1	3.4	3.7
Age at menarche <12 years (%)	25	25	24	24	24
Oral contraceptive user (%)	9	10	10	11	11
Nulliparous (%)	19	19	21	24	28
Parity^b^	2.2	2.2	2.2	2.2	2.2
Age at first birth^b^ (years)	26	26	26	26	26
History of benign breast disease (%)	34	36	36	35	35
Mother or sister with breast cancer (%)	6	7	6	6	6

a Values are the mean or the percent of the person-time over follow-up from 1989 to 1999, standardized to the age distribution of the study population.

b Parity and age at first birth were calculated among parous women only.

). Other risk factors for breast cancer, including age, height, age at menarche, OC use, parity, age at first birth, history of benign breast disease, and family history of breast cancer were unrelated to activity.

Total physical activity was unrelated to risk of breast cancer (Table 2

**Table 2:** Relative risks (RR) of breast cancer (*n*=849) by total activity among premenopausal women in the NHS II cohort, 1989–1999

**NHS**					
	**Person-years (thousands)**	**Cases**	**Age-adjusted RR (95% CI)**	**Multivariate^a^ without BMI RR (95% CI)**	**Multivariate^b^ with BMI RR (95% CI)**
Total activity (MET-h week^−1^)					
<3	106.1	95	1.00	1.00	1.00
3–8.9	217.5	210	1.07 (0.84–1.37)	1.05 (0.82–1.34)	1.05 (0.82–1.33)
9–17.9	225.5	197	0.99 (0.77–1.26)	0.96 (0.75–1.23)	0.95 (0.74–1.21)
18–26.9	140.2	131	1.08 (0.83–1.41)	1.05 (0.80–1.37)	1.03 (0.79–1.35)
⩾27	244.8	216	1.10 (0.86–1.40)	1.07 (0.84–1.36)	1.04 (0.82–1.33)
*P* for linear trend			0.57	0.69	0.86

a Adjusted for age, height, alcohol intake, age at menarche, age at first birth, oral contraceptive use, history of benign breast disease, and mother or sister with breast cancer.

b Adjusted for the factors listed above plus BMI.

). In the multivariate-adjusted analyses, women engaging in ⩾27 MET-h week^−1^ had an RR of 1.04 (95% CI 0.82–1.33) compared to those in the <3 MET-h week^−1^ category. Adjustment for the risk factors, including BMI, only slightly attenuated the RRs adjusted only for age. Results were also unchanged when we included BMI at age 18 years and weight change since age 18 years, instead of current BMI, in the multivariate model, or when we included time since last birth instead of parity and age at first birth (data not shown).

We also evaluated specific low-intensity (walking) and high-intensity (running or jogging) activities (Table 3

**Table 3:** Relative risks (RR) of breast cancer by time spent walking and by time spent running or jogging among premenopausal women in the NHS II cohort, 1989–1999

	**Person-years (thousands)**	**Cases**	**Age-adjusted RR (95% CI)**	**Multivariate^a^ RR (95% CI)**
*Walking*				
<20 min week^−1^	166.2	126	1.00	1.00
20–59 min week^−1^	239.3	228	1.16 (0.93–1.44)	1.17 (0.94–1.46)
1–1.9 h week^−1^	251.9	241	1.20 (0.97–1.49)	1.20 (0.97–1.50)
2–3.9 h week^−1^	157.7	160	1.27 (1.00–1.60)	1.23 (0.97–1.57)
⩾4 h week^−1^	119.0	94	1.07 (0.82–1.40)	1.07 (0.81–1.40)
*P* for linear trend			0.74	0.80

*Running or jogging*				
None	760.4	701	1.00	1.00
<1 h week^−1^	107.9	92	0.94 (0.76–1.17)	0.92 (0.74–1.15)
1–1.9 h week^−1^	36.5	37	1.20 (0.48–1.19)	1.15 (0.83–1.61)
⩾2 h week^−1^	29.3	19	0.76 (0.48–1.19)	0.71 (0.45–1.12)
*P* for linear trend			0.20	0.10

a Adjusted for age, BMI, height, alcohol intake, age at menarche, age at first birth, oral contraceptive use, history of benign breast disease, mother or sister with breast cancer, and MET-h week^−1^ from other activities.

). Walking was unrelated to breast cancer risk (RR=1.07, 95% CI 0.81–1.40, for ⩾4 h week^−1^ *vs* <20 min/wk), while women who ran or jogged for ⩾2 h week^−1^ had a nonsignificantly lower risk (RR=0.71, 95% CI 0.45–1.12, *P* for linear trend=0.10) compared to those who did not engage in either activity. Running and jogging were reported by only 6 and 10%, respectively, of the women in this study, providing modest power for analysis.

We examined the relation between total physical activity and risk of breast cancer within strata of several risk factors (Table 4

**Table 4:** Relative risks (RR) of breast cancer by total activity, stratified by BMI and by oral contraceptive (OC) use, among premenopausal women in the NHS II cohort, 1989–1999

	**BMI <25 kg m^−2^**			**BMI 25–29.9 kg m^−2^**			**BMI ⩾30 kg m^−2^**		
	**Person years (thousands)**	**Cases**	**Multivariate^a^ RR (95% CI)**	**Person-years (thousands)**	**Cases**	**Multivariate^a^ RR (95% CI)**	**Person-years (thousands)**	**Cases**	**Multivariate^a^ RR (95% CI)**
**Total Activity (MET-h/week^−1^)**									
<3	56.7	52	1.00	24.1	20	1.00	24.4	22	1.00
3–8.9	123.1	120	1.06 (0.7601.47)	50.8	49	1.12 (0.66–1.88)	42.4	40	0.95 (0.56–1.60)
9–7.9	137.6	120	0.95 (0.69–1.32)	51.4	50	1.14 (0.67–1.92)	35.4	27	0.76 (0.43–1.34)
18–26.9	90.0	88	1.03 (0.77–1.52)	31.0	33	1.28 (0.73–2.23)	18.5	10	0.56 (0.26–1.18)
⩾27	170.6	144	1.04 (0.72–1.36)	47.9	36	0.98 (0.57–1.70)	24.9	35	1.53 (0.89–2.63)
*P* for linear trend			0.89			0.57			0.04
	**OC use=never**			**OC use=past**			**OC use=current**		
**Total Activity (MET-h/week^−1^)**									
<3	16.0	11	1.00	78.1	79	1.00			
3–8.9	32.5	28	1.23 (0.63–2.56)	160.8	158	0.94 (0.72–1.23)	28.9	25	1.00^b^
9–17.9	33.1	33	1.48 (0.74–2.95)	165.7	140	0.81 (0.61–1.07)	22.6	21	1.07 (0.59–1.92)
18–26.9	20.5	20	1.50 (0.71–3.16)	102.1	100	0.95 (0.71–1.28)	15.2	10	0.75 (0.35–1.57)
⩾27	36.1	26	1.19 (0.58–2.42)	173.4	175	1.04 (0.79–1.36)	29.5	14	0.59 (0.30–1.16)
*P* for linear trend			0.80			0.46			0.14

a All RRs were adjusted for age, BMI, height, alcohol intake, age at menarche, parity, age at first birth, history of benign breast disease, and mother or sister with breast cancer. Analyses stratified by BMI were additionally adjusted for OC use.

b Reference category is <9 MET-h week^−1^ due to small numbers (four cases and 9038 person-years) in the <3 MET-h week^−1^ category.

). Among women with a BMI ⩾30 kg m^2^, we observed a significant positive dose–response relation (*P*=0.04), although the RR in the highest activity category of ⩾27 MET-h week^−1^ was not significant (RR=1.53, 95% CI 0.89–2.63). Activity was unrelated to breast cancer risk at lower levels of BMI. A test for interaction between activity (five categories) and BMI (<30, ⩾30 kg m^−2^) was statistically significant (*P*=0.02). Among current OC users, higher activity was associated with a lower risk of breast cancer (RR=0.59, 95% CI 0.30–1.16 for ⩾27 *vs* <9 MET-h week^−1^, *p* for linear trend=0.14), although a test for interaction between activity (five categories) and OC use (never/past, current) was not statistically significant (*P*=0.08). An inverse association was evident among both shorter (<5 years) and longer (⩾10 years) duration users, although power to assess these relations was low. Also, there was no indication of an inverse association for physical activity once OC use ended. Among the past users who stopped taking OCs within the last 5 years, the relative risk was 1.20 (95% CI 0.57–2.49) for ⩾27 *vs* <3 MET-h week^−1^. In other stratified analyses, associations between physical activity and risk of breast cancer remained null when limited to women <40 or ⩾40 years of age or to nulliparous or parous women, and in strata of smoking and alcohol intake (data not shown).

## DISCUSSION

In these prospective data among premenopausal women, we observed no overall association between recent physical activity and risk of invasive breast cancer. However, when evaluating specific activities we observed a nonsignificant reduction in risk of some 30% among women who run or jog for 2 or more hours per week, but no reduction in risk among women who walk regularly. Within strata of risk factors, we observed no protection among lean women and a significant trend of increased risk with higher activity levels among obese premenopausal women. We also observed a suggestion of reduced risk among current users of OCs that was independent of duration of use.

These data on physical activity have been assessed using an instrument that was validated in the current population and shown to classify women reasonably well. Using the same activity assessment in parallel studies among slightly older women we have shown that greater activity predicts reduced risk of diabetes (Hu *et al*, 1999), heart disease (Manson *et al*, 1999), stroke (Hu *et al*, 2000), colon cancer (Martinez *et al*, 1999), osteoporotic fractures (Feskanich *et al*, 2002), and reduced weight gain after smoking cessation (Kawachi *et al*, 1996). In the NHS II, higher physical activity predicts reduced ovulatory infertility (Rich-Edwards *et al*, 2002), and is strongly related to lower BMI. Other lifestyle exposures considered as confounders and effect modifiers in this prospective study have also shown high levels of validity in self-report (Troy *et al*, 1995; Hunter *et al*, 1997). High follow-up and confirmation rates of cancer among these cohort participants further reduces the potential for bias in these results.

The finding for type of activity and reduction in breast cancer risk may offer some insight into the potential source of variation in the findings among the studies to date. In this cohort, although the results for strenuous activities of running and jogging were of borderline significance for 2 or more hours per week, power was limited because only 3% of women reported running and jogging at this level.

As the prevalence of obesity continues to rise in the US (Flegal *et al*, 2002), the observation that among premenopausal women higher activity among obese women is directly related to risk may offer an explanation for the lack of an overall reduction in risk within this population. Previous studies observing a strong inverse association among premenopausal women have, on average, been conducted among women with lower BMI. Verloop *et al* (2000), using a case–control design, evaluated 998 cases among women younger than 55 years of age in the Netherlands, with a mean BMI of 24.2 kg m^−2^. They observed a stronger protection for lifetime recreational activity among lean women (RR=0.57; 95% CI 0.40–0.82) and no reduction in risk among women with BMI greater than 24.5 kg m^−2^. (RR=0.92; 95% CI 0.65–1.29). Bernstein *et al*, using a case-control design, evaluated 545 cases diagnosed among women less than age 40 years and observed greatest protection among women with sustained activity from adolescence through early adult years. In that population 75% of women had a BMI below 25 kg m^−2^, substantially leaner than the average US population in the late 1980's when 25.8% of women 30 to 39 years of age were obese (>30 kg m^−2^) (Flegal *et al*, 2002). Coogan *et al* (1997) also reported a stronger association among leaner women, but like several other case–control studies, the test for interaction was not significant. Among postmenopausal women, several studies have noted that the inverse association with higher activity is only evident among women who have not gained substantial weight during their adult years (Carpenter *et al*, 1999; Shoff *et al*, 2000). Several other case–control studies limited to, or predominantly including, premenopausal breast cancer have often failed to show an inverse association with level of recent or sustained physical activity and report no variation in the association by level of BMI (Chen *et al*, 1997; Gammon *et al*, 1998; Friedenreich *et al*, 2001). In the only prospective study stratifying results according to BMI, Thune *et al* (1997) followed women in Norway and observed a significant inverse trend in risk among lean women (*P* trend =0.02 among women less than 22.8 kg m^−2^), but not among average weight or overweight women (*P* trend=0.90 and 0.36 respectively). 

Physical activity may reduce risk of breast cancer through several possible mechanisms including change in hormonal exposure, change in immune function, and (in postmenopausal women) reduction in weight gain. To date, immune function has not been related to risk of breast cancer and hence this potential mechanism is not discussed further. Very high levels of activity may reduce the frequency of ovulation or the length of the luteal phase (Bernstein *et al*, 1987), thus reducing exposure to oestradiol and progesterone. Some evidence suggests that physical activity insufficient to cause anovulation may also reduce oestrogen levels (Bullen *et al*, 1985). Among premenopausal women, increasing BMI is associated with lower risk of breast cancer (van den Brandt *et al*, 2000), and this has been hypothesised to result from anovulation induced by insulin resistance, which is reduced by physical activity. The increase in risk associated with greater physical activity among obese premenopausal women could then be due to a reduction in insulin resistance and increased frequency of ovulation. Indirect evidence supporting this hypothesis comes from the current prospective cohort study in which we have observed that obese women have more irregular menstrual cycles, and greater risk of infertility (Rich-Edwards *et al*, 1994), and that regular activity is associated with reduced anovulatory infertility (Rich-Edwards *et al*, 2002). Furthermore, in this cohort, irregular menstrual cycles and anovulatory infertility are associated with reduced risk of premenopausal breast cancer (Garland *et al*, 1998). The suggestion of a benefit among women who currently use OCs would be compatible with a lack of adverse effect by reducing ovulation (users are all anovulatory) combined with a benefit independent of ovulation.

In conclusion, these data among premenopausal women suggest that there is no overall association between activity and risk of breast cancer. However, benefits from high-intensity activity such as running and jogging cannot be excluded and require further evaluation. Our findings also suggest that the effect of physical activity could be substantially modified by the underlying degree of adiposity. The potential interactions between physical activity, adiposity, and current use of oral contraceptives require additional study.

## References

[bib1] Ainsworth B, Haskell W, Leon A, Jacobs Jr D, Montoye H, Sallis J, Paffenbarger Jr, R (1993) Compendium of physical activities: classification of energy costs of human physical activities. Med Sci Sports Exerc 25: 71–80 8292105 10.1249/00005768-199301000-00011

[bib2] Bernstein L, Ross R, Lobo R, Hanisch R, Krailo M, Henderson B (1987) The effects of moderate physical activity on menstrual cycle patterns in adolescence: implications for breast cancer prevention. Br J Cancer 55: 681–685 3620313 10.1038/bjc.1987.139 PMC2002035

[bib3] Bullen BA, Skrinar GS, Beitins IZ, von Mering G, Turnbull BA, McArthur JW (1985) Induction of menstrual disorders by strenuous exercise in untrained women. N Engl J Med 312: 1349–1353 3990734 10.1056/NEJM198505233122103

[bib4] Carpenter CL, Ross RK, Paganini-Hill A, Bernstein L (1999) Lifetime exercise activity and breast cancer risk among post-menopausal women. Br J Cancer 80 (11): 1852–1858 10468309 10.1038/sj.bjc.6690610 PMC2374273

[bib5] Chen CL, White E, Malone KE, Daling JR (1997) Leisure-time physical activity in relation to breast cancer among young women (Washington, United States). Cancer Causes Control 8: 77–84 9051326 10.1023/a:1018439306604

[bib6] Coogan P, Newcomb P, Clapp R, Trentham-Dietz A, Baron J, Longnecker M (1997) Physical activity in usual occupation and risk of breast cancer (United States). Cancer Causes Control 8: 626–631 9242479 10.1023/a:1018402615206

[bib7] Cox DR (1972) Regression models and life-tables [with discussion]. J Royal Stat Soc (B) 34: 187–220

[bib8] Feskanich D, Willett W, Colditz G (2002) Walking and leisure-time activity and risk of hip fracture in postmenopausal women. JAMA 288 (18): 2300–2306 12425707 10.1001/jama.288.18.2300

[bib9] Flegal KM, Carroll MD, Ogden CL, Johnson CL (2002) Prevalence and trends in obesity among US adults, 1999–2000. JAMA 288 (14): 1723–1727 12365955 10.1001/jama.288.14.1723

[bib10] Friedenreich CM, Courneya KS, Bryant HE (2001) Influence of physical activity in different age and life periods on the risk of breast cancer. Epidemiology 12 (6): 604–612 11679785 10.1097/00001648-200111000-00005

[bib11] Gammon MD, John EM, Britton JA (1998) Recreational and occupational physical activities and risk of breast cancer. J Natl Cancer Inst 90: 100–117 9450570 10.1093/jnci/90.2.100

[bib12] Garland M, Hunter DJ, Colditz GA, Manson JE, Stampfer MJ, Spiegelman D, Speizer F, Willett WC (1998) Menstrual cycle characteristics and history of ovulatory infertility in relation to breast cancer risk in a large cohort of women. Am J Epidemiol 147: 636–643 9554602 10.1093/oxfordjournals.aje.a009504

[bib13] Hu F, Sigal R, Rich-Edwards J, Colditz G, Solomon C, Willett W, Speizer F, Manson J (1999) Walking compared with vigorous physical activity and risk of type 2 diabetes in women: a prospective study. JAMA 282: 1433–1439 10535433 10.1001/jama.282.15.1433

[bib14] Hu F, Stampfer M, Colditz G, Ascherio A, Rexrode K, Willett W, Manson J (2000) Physical activity and risk of stroke in women. JAMA 283: 2961–2967 10865274 10.1001/jama.283.22.2961

[bib15] Hunter DJ, Manson JE, Colditz GA, Chasan-Taber L, Troy L, Stampfer MJ, Speizer FE, Willett WC (1997) Reproducibility of oral contraceptive histories and validity of hormone composition reported in a cohort of US women. Contraception 56: 373–378 9494771 10.1016/s0010-7824(97)00172-8

[bib16] International Agency for Research on Cancer (2002) Weight Control and Physical Activity. Lyon: International Agency for Research on Cancer

[bib17] Kawachi I, Troisi R, Rotnitzky A, Coakley E, Colditz G (1996) Can physical activity minimize weight gain in women after smoking cessation? Am J Public Health 86: 999–1004 8669525 10.2105/ajph.86.7.999 PMC1380442

[bib18] Manson J, Hu F, Rich-Edwards J, Colditz G, Stampfer M, Willett W, Speizer F, Hennekens C (1999) A prospective study of walking as compared with vigorous exercise in the prevention of coronary heart disease in women. N Engl J Med 341: 650–658 10460816 10.1056/NEJM199908263410904

[bib19] Martinez M, Heddens D, Earnest D, Bogert C, Roe D, Einspahr J, Marshall J, Alberts D (1999) Physical activity, body mass index, and prostaglandin E2 levels in rectal mucosa. J Natl Cancer Inst 91: 950–953 10359547 10.1093/jnci/91.11.950

[bib20] Rich-Edwards JW, Goldman MB, Willett WC, Hunter DJ, Stampfer MJ, Colditz GA, Manson JE (1994) Adolescent body mass index and ovulatory infertility. Am J Obstet Gynecol 171: 171–177 8030695 10.1016/0002-9378(94)90465-0

[bib21] Rich-Edwards JW, Spiegelman D, Garland M, Hertzmark E, Hunter DJ, Colditz GA, Willett WC, Wand H, Manson JE (2002) Physical activity, body mass index, and ovulatory disorder infertility. Epidemiology 13 (2): 184–190 11880759 10.1097/00001648-200203000-00013

[bib22] Rimm EB, Stampfer MJ, Colditz GA, Chute CG, Litin LB, Willett WC (1990) Validity of self-reported waist and hip circumferences in men and women. Epidemiology 1: 466–473 2090285 10.1097/00001648-199011000-00009

[bib23] Rockhill B, Willett WC, Hunter DJ, Manson JE, Hankinson SE, Spiegelman D, Colditz GA (1998) Physical activity and breast cancer risk in a cohort of young women. Natl Cancer Inst 90: 1155–1160 10.1093/jnci/90.15.1155 9701365

[bib24] Shoff SM, Newcomb PA, Trentham-Dietz A, Remington PL, Mittendorf R, Greenberg ER, Willett WC (2000) Early-life physical activity and postmenopausal breast cancer: effect of body size and weight change. Cancer Epidemiol Biomarkers Prev 9 (6): 591–595 10868694

[bib25] Stampfer MJ, Willett WC, Speizer FE, Dysert DC, Lipnick R, Rosner B, Hennekens CH (1984) Test of the National Death Index. Am J Epidemiol 119: 837–839 6720679 10.1093/oxfordjournals.aje.a113804

[bib26] Thune I, Brenn T, Lund E, Gaard M (1997) Physical activity and the risk of breast cancer. N Engl J Med 336: 1269–1275 9113929 10.1056/NEJM199705013361801

[bib27] Troy LM, Hunter DJ, Manson JE, Colditz GA, Stampfer MJ, Willett WC (1995) The validity of recalled height and past weight among younger women. Int J Obes 19: 570–572 7489028

[bib28] van den Brandt PA, Spiegelman D, Yaun SS, Adami HO, Beeson L, Folson AR, Fraser G, Goldbohm RA, Graham S, Kushi L, Marshall JR, Miller AB, Rohan T, Smith-Warner SA, Speizer FE, Willett WC, Wolk A, Hunter DJ (2000) Pooled analysis of prospective cohort studies on height, weight, and breast cancer risk. Am J Epidemiol 152: 514–527 10997541 10.1093/aje/152.6.514

[bib29] Verloop J, Rookus MA, van der Kooy K, van Leeuwen FE (2000) Physical activity and breast cancer risk in women aged 20–54 years. J Natl Cancer Inst 92 (2): 128–135 10639514 10.1093/jnci/92.2.128

[bib30] Wolf A, Hunter D, Colditz GA, Manson JE, Stampfer MJ, Corsano K, Corsano KA, Rosner B, Kriska A, Willett WC (1994) Reproducibility and validity of a self-administered physical activity questionnaire. Int J Epidemiol 23: 991–999 7860180 10.1093/ije/23.5.991

